# Explainable Machine Learning Models for Brain Diseases: Insights from a Systematic Review

**DOI:** 10.3390/neurolint16060098

**Published:** 2024-10-29

**Authors:** Mirko Jerber Rodríguez Mallma, Luis Zuloaga-Rotta, Rubén Borja-Rosales, Josef Renato Rodríguez Mallma, Marcos Vilca-Aguilar, María Salas-Ojeda, David Mauricio

**Affiliations:** 1 Facultad de Ingeniería Industrial y de Sistemas, Universidad Nacional de Ingeniería, Lima 15333, Peru; mjrodriguezm@uni.pe (M.J.R.M.); zuloaga_luis@uni.edu.pe (L.Z.-R.);; 2 Instituto de Radiocirugía del Perú, Clínica San Pablo, Lima 150140, Peru; 3 Facultad de Artes y Humanidades, Universidad San Ignacio de Loyola, Lima 15024, Peru; 4 Facultad de Ingeniería de Sistemas e Informática, Universidad Nacional Mayor de San Marcos, Lima 15081, Peru; dmauricios@unmsm.edu.pe

**Keywords:** explainable artificial intelligence (XAI), machine learning (ML), healthcare, diagnosis, prognosis, risk, brain diseases

## Abstract

In recent years, Artificial Intelligence (AI) methods, specifically Machine Learning (ML) models, have been providing outstanding results in different areas of knowledge, with the health area being one of its most impactful fields of application. However, to be applied reliably, these models must provide users with clear, simple, and transparent explanations about the medical decision-making process. This systematic review aims to investigate the use and application of explainability in ML models used in brain disease studies. A systematic search was conducted in three major bibliographic databases, Web of Science, Scopus, and PubMed, from January 2014 to December 2023. A total of 133 relevant studies were identified and analyzed out of a total of 682 found in the initial search, in which the explainability of ML models in the medical context was studied, identifying 11 ML models and 12 explainability techniques applied in the study of 20 brain diseases.

## 1. Introduction

The recent advancements in Artificial Intelligence (AI) through the improvement of various techniques, algorithms, and models, particularly Machine Learning (ML) ones, have shown significant potential in revolutionizing medical practice [1,2,3,4,5]. However, for these models to be applied reliably in healthcare, they must provide users with clear, simple, and transparent explanations about the medical decision-making process. This reassures the audience about the reliability of these models. Many ML models used in the medical field are challenging to understand, and their results could be perceived as unreliable [6]. Therefore, an adequate understanding of ML models has become a condition that must be resolved before widespread use in the medical field.

Two main concepts help in fostering an adequate understanding and trustability of ML models: explainability, which aims to build confidence in using AI systems to ensure their results are safe and reliable [7], and interpretability, which can be seen as a broader component of explainability that provides a high-level view of the model without going into specific details [8]. Even more, concerns about the explainability of algorithms, models, or systems are not recent, as shown by Confalonieri et al. [9], who carried out a historical study of the topic and found that there are different notions, perspectives, understandings, and uses of explainability in medical decision-making systems. Therefore, solving the problem of the explainability of AI models is critical to avoid the possible negative consequences of using automated systems that do not provide reliable explanations of their results [6].

The discussion on explainability in medical practice is also approached from an ethical and legal point of view, as shown by Astromskė et al. [10], who reviewed these two aspects in the automation of medical decision-making processes, highlighting, for example, within the ethical aspect, the right of patients to an explanation of the results obtained, and in the legal aspect, the limits and requirements of explainability. Also, Ploug et al. [11] suggest that patients should have the right to question an AI system for possible inaccuracies that the results may present caused by (1) the use of imprecise personal data, (2) biases inherent to the system, (3) system performance, and (4) responsibility of the system versus the health professional. Therefore, it is necessary to establish a regulatory framework aligned with ethical, legal, and social demands that guarantee a compelling application and use of AI systems in the medical area, as also suggested by Currie and Hawk [12].

While the explanation and interpretation of the model’s output are required to improve the use of these systems in various clinical applications [13], the implications in interpretability—the understanding of how a system operates or behaves—are critical and highly recommended to evaluate how the models will perform in real-world scenarios to help gain trust in these kinds of systems in medical environments [14]. Therefore, from the transparency point of view, two types of ML models that support explainability are currently being used: (1) explainable ML models that are transparent by default and (2) non-explainable ML models that have complex architectures that, in turn, make them not very transparent and difficult to understand. However, the lack of transparency of non-explainable ML models can be solved by applying any Explainability Technique (ET) that makes it possible to “open” said models to understand, interpret, and explain them efficiently. These ETs allow building ML systems that meet two objectives: (1) making predictions with the maximum possible precision and (2) making these predictions as understandable as possible so that their use generates confidence.

In recent years, a series of investigations have been published that highlight the importance of the explainability of AI algorithms to clear up doubts among medical professionals, generate trust in patients, and create the conditions that allow the actual use of these algorithms to be incorporated into medical practice [15,16,17]. For example, Fraccaro et al. [18] conducted a study to diagnose adult macular degeneration, and McRae et al. [19] diagnosed cardiovascular diseases, and in both studies, an explainable ML approach was applied. Other studies, such as that of Angius et al. [20], who predict the possibility of infection in patients with leukemia and that of Lu et al. [21], who study the diagnosis and prognosis of patients with COVID-19, make use of non-explainable ML models along with ETs. All these studies show the need to consolidate all the knowledge accumulated to date about explainable and non-explainable ML models and the ETs used in different applications in the medical area to make their practical use possible.

For all the above, and taking into account that brain diseases are considered a global health problem [22], where many of their risk factors are still unknown but associated with multiple conditions such as biological, demographic, environmental, genetic, lifestyle, and pharmacological ones [23,24], and with opportunities and challenges to be resolved such as lacking metrics or tools for qualify brain health, little knowledge about the mechanisms of brain function and dysfunction, few effective approaches to prevent and treat brain dysfunctions [25], and with novel approaches to help in their diagnosis, prognosis and treatment [26,27], including the use of AI methods [1,28,29,30,31,32,33], this work focuses on answering the following central question: What progress has been made in the study of the explainability of ML models used in the medical area and applied to brain diseases? Therefore, this work’s main contributions are as follows:

- Provide an overview of the application and use of explainable AI in the medical area, specifically Explainable Machine Learning (EML), reviewing aspects such as ML models, ETs, and applications in brain diseases in the last ten years.
- Provide the reader with a wide range of bibliographic references that they can use to understand and investigate in more detail the topic of explainable AI in the medical area, specifically its use in brain diseases.

This article is organized into six sections. Section 2 describes the explainability of ML models in the medical area. Section 3 presents and describes the research methodology used. Section 4 analyzes the results resulting from the selected literature. Section 5 discusses the study. Finally, the conclusions and limitations are presented in Section 6 and Section 7, respectively.

## 2. Explainability of ML Models in Healthcare

### 2.1. Explainability

The definition provided by the Cambridge Dictionary [34] states that the term “explanation” means “the details or reasons that someone gives to make something clear or easy to understand”. This gives us a general idea of what is understood when we refer to the term explainability. In simple terms, explainability, in the context of AI, can be understood as the characteristic of AI algorithms that explains “how” and “why” a given result or prediction was obtained. It is essential to highlight that current interest in the study and application of explainability has been resumed; furthermore, a new field of research called Explainable Artificial Intelligence (XIA) has been created, a term coined in 2004 by Lent et al. [35] to refer to the ability of systems to explain the behavior of AI components in simulated games.

Today, there is a growing need to include mechanisms that allow the application of explainability approaches in AI algorithms or any automated decision-making system. For example, in the European Union, since 2016, there is a regulation known as the General Data Protection Regulation (GDPR) [36], which establishes that any individual subject to an automated decision-making system has the right to demand an explanation of why a result was reached as a consequence of the use of any automated mechanism; therefore, the entity responsible for the automated decision-making system must implement the mechanisms that ensure that the results obtained are fully transparent for the end users to use.

It is essential to remember that the term explainability has various definitions. Furthermore, its terminology would be poorly defined, and other terms, such as interpretability and/or transparency, are often used and accepted as synonyms [37]. While explainability is mainly associated with the notion of explanation and includes the ability to provide detailed and understandable reasoning for specific decisions, interpretability can be understood as a high-level view of the model without going into specific details and involves not only understanding but also describing how a system operates or behaves [8]. Therefore, intending to include all studies that use any or both terminologies in this systematic review, this article uses the definition of explainability proposed by Adadi and Berrada [38], which states that “it is a field of research that aims to make the results of AI systems more understandable for humans” and the definition of interpretability used by Barreto Arrieta et al. [37], which states that “it is the ability to explain or to provide the meaning in understandable terms to a human”.

### 2.2. Explainability in Medicine

In medicine, explainability is a very important characteristic that must accompany the medical results produced by an algorithm or AI system, especially when these systems tend to return “raw results” that are difficult to understand for non-expert users [39].

To date, various AI methods and systems have been proposed for the diagnosis and/or prognosis of disease and medical conditions such as COVID-19 [40,41], depression [42,43], mortality [44], leukemia [20], migraine [45], autism [46], etc. However, its application is still incipient because many algorithms behind these AI systems are not transparent, generating “mistrust” about their results [47]. Therefore, a barrier would still exist, and the adoption of AI systems would be avoided.

An expected application of explainability in the medical area would imply, for example, that a given medical diagnosis based on an AI system must include detailed and understandable explanations of how a diagnosis was reached and why it is the best possible choice according to the set of data available in a particular medical situation.

### 2.3. Explainability Approaches

From the point of view of the types of ML models considered for this study, we can identify “white box” ML models, which are explainable by default, and “black box” ML models, which are not explainable; this is based on the approach proposed by Loyola-González [48].

**Figure 1:**
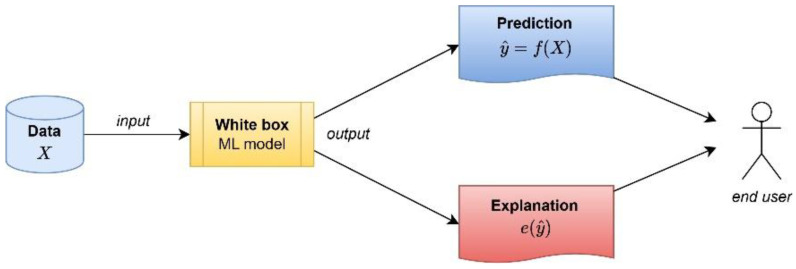
The process flow of “white box” models.

In this article, we understand that “white box” models are easy to understand and whose results can be easily explained without the need for additional complex mathematical techniques [49]. Figure 1 shows a diagram of a “white box” model where the results obtained from the input data are composed of (1) the forecast and (2) the explanation of the forecast without the need for any other additional technique. Furthermore, some studies, such as the one of Rudin [45], recommend its use as the first option when explainability is a must.

**Figure 2:**
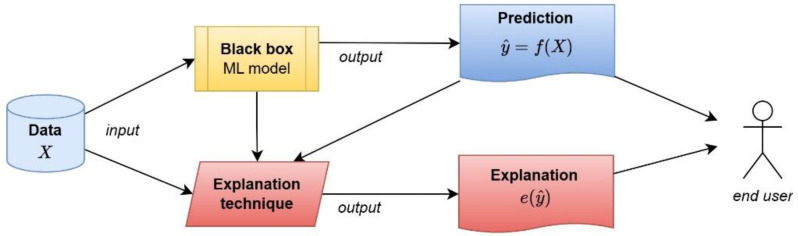
The process flow of “black box” models.

On the other hand, an ML model is considered a “black box” when it is established through a function that is very difficult to understand or that is proprietary [50]. Therefore, these models are difficult to understand and usually require additional mechanisms to make them transparent, as shown in Figure 2, which requires an additional explainability method or technique.

Both “white box” and “black box” ML models were considered for this study.

## 3. Materials and Methods

This study’s literature review process was carried out using the recommendations and guidelines of the document called Preferred Reporting Items for Systematic Reviews and Meta-Analyses (PRISMA) [51,52], and it was registered on the Open Science Framework (OSF) repository (DOI: 10.17605/OSF.IO/9DNUK). Also, both the PRISMA 2020 Abstract checklist and the PRISMA 2020 checklist can be found in Table S1 and Table S2 respectively. Furthermore, taking into account that these types of studies are used for the synthesis and analysis of a wide range of research materials or to provide conceptual clarity about a topic [53], our study, through research questions, sought to inventory the different EML models and ETs used in the medical area, as well as examine and analyze specific applications in the diagnosis, prognosis, risks, or treatment of brain diseases.

### 3.1. Research Questions

To answer the central research question mentioned in the *Introduction* section, the following sub-questions were defined:

- RQ1: Which ML models include explainability and are applied to brain diseases in the medical area?
- RQ2: Which ETs for ML models are applied to brain diseases?
- RQ3: What is the use of EML models applied to brain diseases?
  ○ RQ3.1: What brain diseases are the most studied?
  ○ RQ3.2: What disease study types are the most relevant?

### 3.2. Search Strategy

To answer the research sub-questions, a systematic search was carried out for articles from journals indexed in the Web of Science, Scopus, and PubMed databases between January 2014 and December 2023.

The search was performed using the search strings shown in Table 1.

### 3.3. Inclusion and Exclusion Criteria

Table 2 shows the inclusion criteria (IC) and exclusion criteria (EC) used in this study to select articles.

### 3.4. Quality Assessment

The quality of the articles was evaluated using the Newcastle–Ottawa Scale (NOS), a well-known and widely used method to evaluate the relevance of research works [56].

**Figure 3:**
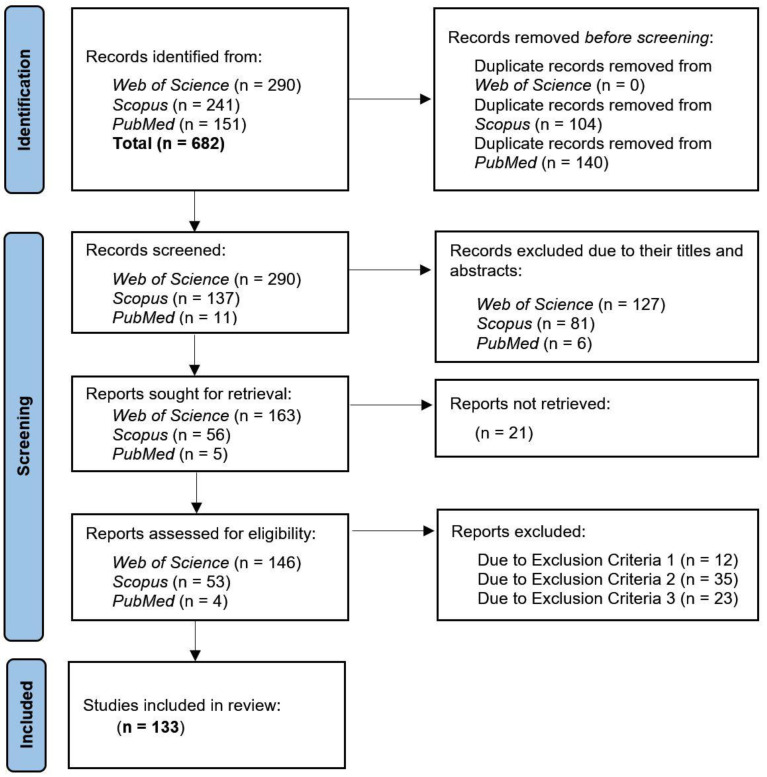
Flow for the identification of relevant studies based on PRISMA.

Figure 3 shows the search process used in this study, based on applying the PRISMA flowchart in its 2020 version [57]. As can be seen, after applying the search strings in Table 1 (in which the exclusion criteria EC4 and EC5 from Table 2 were already considered), a total of 682 potential articles were obtained, of which 244 repeated were removed; then, an additional 214 articles were removed due to titles and/or abstracts that were not relevant, and another 21 articles that could not be obtained were removed. Then, the remaining 203 articles were exhaustively reviewed, of which 70 were removed due to the exclusion criteria EC1, CE2, and CE3 to finally obtain 133 articles that were part of this systematic review study.

## 4. Results

After applying the PRISMA methodology, 133 relevant articles were identified and included in this systematic review.

**Figure 4:**
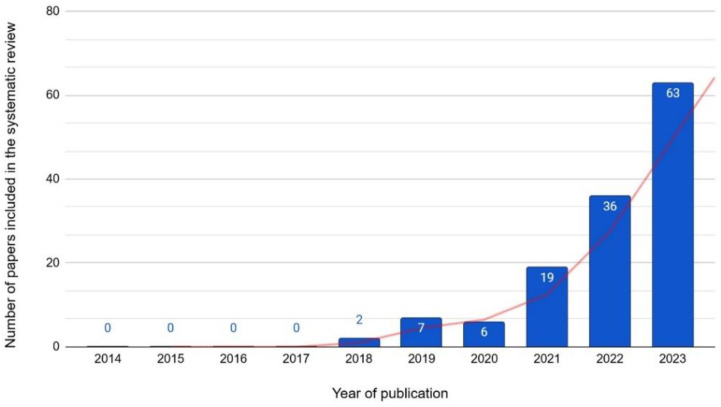
Number of relevant articles per year (2014–2023).

Figure 4 reveals a growing trend in the study and publication of articles subject to our analysis by the scientific community; however, real growth is only evident from 2018 onwards.

The results obtained for each research sub-question are detailed below.

### 4.1. RQ1: Which ML Models Include Explainability and Are Applied to Brain Diseases in the Medical Area?

Eleven types of ML models applied to brain diseases were identified (see Table 3). These ML models were classified according to the proposals of Faouzi and Colliot [54], and An et al. [55], who identified a list of ML models most frequently used in the medical area. 

**Figure 5:**
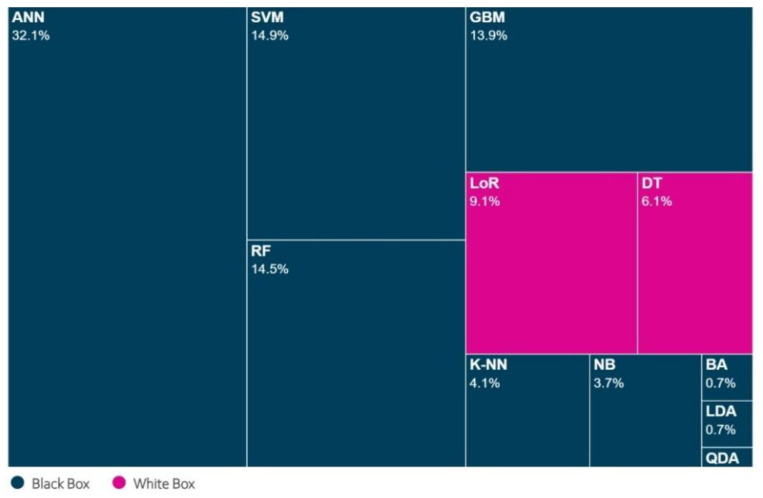
Percentage usage of ML models.

Figure 5 shows that ANN, SVM, RF, and GBM are the most commonly used ML models, appearing in 75% of studies. Furthermore, only two “white box” ML models have been identified: DT and LoR.

### 4.2. RQ2: Which ETs for ML Models Are Applied to Brain Diseases?

Twelve ETs of ML models most frequently used in the study of brain diseases were identified (see Table 4). In addition, the “other” category was identified, which included 27 studies that could not be identified in the classification used for this work [191,192].

**Figure 6:**
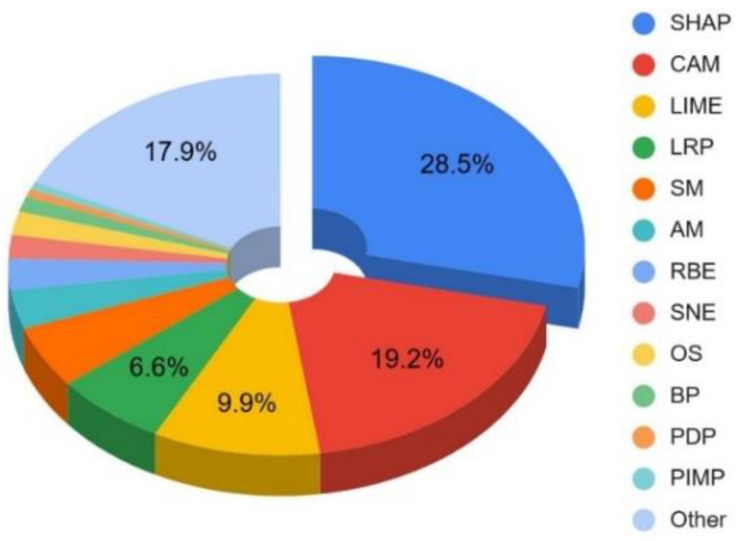
Percentage usage of ETs in ML models.

The most commonly used techniques were SHAP, CAM, and LIME, representing around 58% of all ETs (see Figure 6).

**Figure 7:**
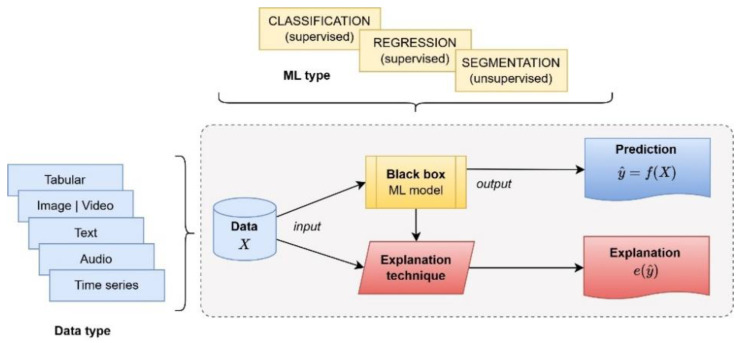
Data types and ML types used in the articles.

In addition, in this section, we also analyzed the type of ML models and data types used by the articles. See Figure 7 as a reference.

**Figure 8:**
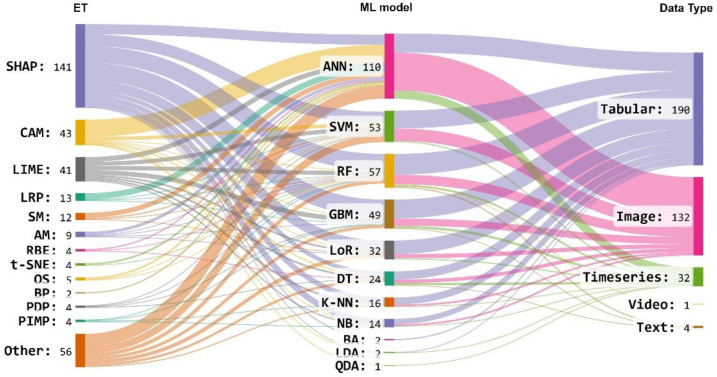
Data types used by the articles in this systematic review.

**Figure 9:**
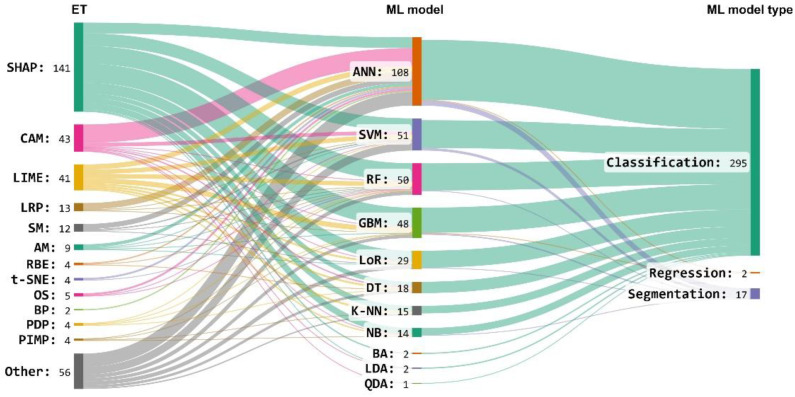
ML types used by the articles in this systematic review.

It was found that ML models mostly use Tabular and Image datatypes (Figure 8), and Classification was the predominant type of ML model (Figure 9).

### 4.3. RQ3: What Is the Use of EML Models Applied to Brain Diseases?

**Figure 10:**
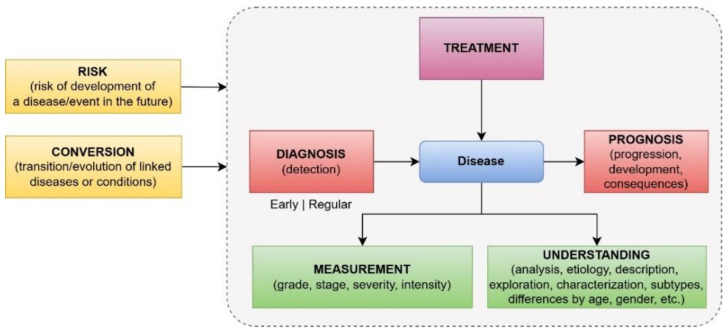
Disease study types used in the medical practice.

To better understand the EML models applied to brain diseases, a classification was made regarding the disease study type in medical practice: diagnosis, prognosis, treatment, measurement, understanding, risk, and conversion. Therefore, the studies have been classified according to this approach (Figure 10).

It is important to highlight that “risk” and “conversion” are approaches that study the disease even before its appearance. The first refers to the risk of developing the disease in the future, and the second refers to the appearance of the disease as a consequence of another previous disease. Although “conversion” can also be understood as a risk of developing a disease, we used a different word/category to explicitly categorize studies where diseases result from another one.

- RQ3.1: What brain diseases are the most studied?

The medical applications and uses identified during the systematic review found that the EML models were mainly applied to 20 diseases or medical conditions, as shown in Table 5.

- RQ3.2: What disease study types are the most relevant?

**Figure 11:**
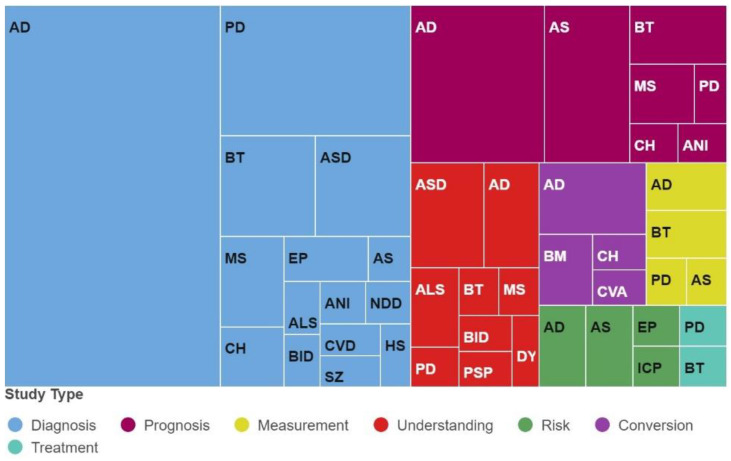
Diseases in the articles grouped by study type.

As shown in Figure 11, most studies applied EML models to the diagnosis (56.25%) and prognosis (18.06%) of brain diseases.

**Figure 12:**
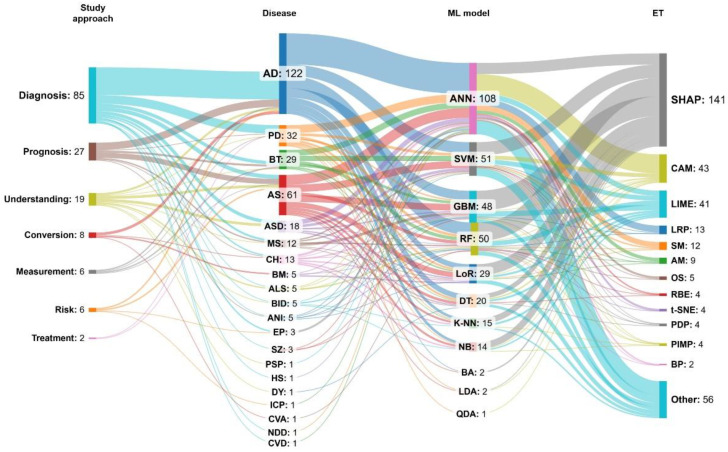
EML applied to brain diseases grouped by study approach.

Finally, most articles used the SHAP technique to explain the ML models. In addition, the most used ML model was the ANN, which was mainly used to study the diagnosis of AD disease (Figure 12).

## 5. Discussion

This study conducted a systematic review of the use of ML models in brain diseases between January 2014 and December 2023, and 682 potential articles were identified, of which 133 were selected and reviewed in detail.

Evidence was found about the growth in the interest of the scientific community in the use of ML models in the medical field, specifically in brain diseases that, in addition to providing precise and optimal results comparable to those obtained by human specialists, could also help them to be explainable and transparent, as shown in Figure 4; this finding correlates with what has been reported in several studies in which the explainability of ML models is considered a crucial aspect for their adoption in medical practice [193], and its inclusion should be mandatory.

The results of this study also show a preference for the use of “black box” ML models compared to “white box” models in the medical field, specifically in their application to brain diseases (see Figure 5). This may be because the formers usually have a high degree of precision; however, they require additional techniques to make them transparent and, therefore, are more complex to implement in medical practice. However, according to several studies such as that of Loyola-González [48], “white box” and “black box” models would have a similar performance, and it depends on the application domain and the data used. Therefore, in the medical field, when “white box” or “black box” models provide similar results in terms of predictability, “white box” models should be preferred because they inherently provide interpretability [50].

The most widely used ML model is ANN, which, being very flexible, can be applied to different data types (see Figure 8). These types of models, specifically CNNs, have been used very successfully in recent years in the analysis of medical images such as X-rays or Magnetic Resonance Imaging (MRI) for the study of diseases in a revolutionary discipline known as Deep Learning (DL), which some authors consider as AI 2.0 [194]. This correlates with the finding that one of the most widely used ETs in the studies that are part of this systematic review is CAM, which is characterized by creating and identifying heat maps in the images analyzed by ML models and which allows explaining the result of the obtained predictions.

The most commonly used ET was SHAP, a method based on the game theory approach applied to virtually all the ML models in this study (Figure 8). This can be explained because this ET is very consistent since it uses all the possible predictions of all the possible combinations of the input data. Another widely used ET was LIME, which, unlike SHAP, is faster in its calculation but is just as popular as SHAP and is frequently found in studies of explainable ML models [195].

It was found that the studies in this research work were applied to a total of 20 brain diseases. Still, most of them focused on the diagnosis and prognosis of AD and PD, two neurodegenerative diseases with the highest recurrence nowadays. The studies of these diseases represent about 50% of all the studies included in this systematic review. The availability of open data about these diseases could explain the predilection for studying these diseases. For example, there is a database named The Alzheimer’s Disease Neuroimaging Initiative (ADNI), which is complete and freely available and contains data from patients with AD (clinical data, images, genetic information, and biomechanical markers). Therefore, we could also deduce that the quantity and quality of available and easily accessible data is not a trivial matter and, on the contrary, becomes an essential resource for developing research that uses ML models and ETs applied to the medical field.

Most of the disease study types used in the articles focus on diagnosis and prognosis; this is expected since the “triad” comprised of diagnosis, prognosis, and treatment is typically considered when approaching the study of diseases [196]. However, researchers also had significant interest in studying other aspects, such as the understanding, conversion, measurement, risk, and treatment of diseases using ML models and ETs, representing 26% of the total articles included in this research.

Even though this work is one of the first systematic reviews to study the use and application of EML models for brain diseases, its main findings correlate with the results of previous and similar works. For instance, there is a clear rising trend in studying the application of interpretability-related concepts in the medical field [8,191,197]. In addition, one of the main ML models used in previous studies was the ANN, specifically the CNN [8,198], which presents several architectures and levels of complexity but is used due to its good performance in analyzing and understanding brain medical images [1,199]. Finally, there was a coincidence in our findings regarding the predilection for using SHAP, LIME, and CAM as the main ETs in medical applications [198,200]. 

## 6. Conclusions

The use of AI methods, techniques, models, and algorithms in the medical field has gradually increased in recent years through the use of a significant number of efficient automated solutions applicable to various real-world clinical problems that even have approval from government institutions such as the Food and Drug Administration (FDA) [201]. Specifically, in the health area, in recent years, thanks to the accumulation of relevant data and the development of more effective algorithms and new methods for explaining their results, the study and understanding of brain diseases have significantly increased, as shown by the results of this systematic review study.

The research community’s efforts are leading to the creation of increasingly sophisticated AI algorithms, without leaving aside the critical aspect of explainability that would allow a widespread adoption, with less rejection and more transparency. This, in turn, would favor a more intensive use of “smart” technologies in the near future.

Finally, the authors consider that to increase the use and encourage the adoption of XAI in medical practice, all automated medical decision-making systems, including AI, must necessarily include the explainability mechanism as a central and mandatory component in their implementation. This mechanism will allow users to know, understand, and comprehend the medical results produced by any automated system.

## 7. Limitations

This study was limited to the Scopus, Web of Science, and PubMed repositories since they are the most relevant ones and correspond to the period from January 2014 to December 2023, so it could be extended to other repositories.

Although this systematic review study includes essential information regarding the use of EML models in the medical field, the following limitations are identified that should be expanded in future research:

- The present work did not find results related to the use of Large Language Models (LLM) or Generative AI (GenAI) as a topic related to explainability in the medical field, and it is something that should be studied given the popularity and novelty of these models.
- Our work has not included the study of the ethical and legal aspects of using medical data to design and train ML models and their impact on regulatory systems.

## Figures and Tables

**Table 1:** Search query string and number of results by source.

Source	Search Query
Web of Science	(TS=((“Explainab*” OR “Interpretab*” OR “XAI”) AND (“Artificial Intelligence” OR “Machine Learning” OR “Deep Learning”) AND (“brain” OR “neuro*”) AND (“disease*”)) AND DT==(“ARTICLE”) AND LA==(“ENGLISH”)) AND (EDN==(“WOS.SCI”)) Timespan: 2014-01-01 to 2023-12-31 (Publication Date)
Scopus	TITLE-ABS-KEY((“Explainab*” OR “Interpretab*” OR “XAI”) AND (“Artificial Intelligence” OR “Machine Learning” OR “Deep Learning”) AND (“brain” OR “neuro*”) AND (“disease*”)) AND (PUBYEAR > 2013) AND (PUBYEAR < 2024) AND (LIMIT-TO(DOCTYPE,”ar”)) AND (LIMIT-TO(SUBJAREA,”MEDI”)) AND (LIMIT-TO(LANGUAGE,”English”)) AND (LIMIT-TO(PUBSTAGE,”final”))
PubMed	((“Explainab*”[Title/Abstract] OR “Interpretab*”[Title/Abstract] OR “XAI”[Title/Abstract]) AND (“Artificial Intelligence”[Title/Abstract] OR “Machine Learning”[Title/Abstract] OR “Deep Learning”[Title/Abstract]) AND (“brain”[Title/Abstract] OR “neuro*”[Title/Abstract]) AND “disease*”[Title/Abstract] AND (2014/01/01:2023/12/31[Date-Publication] AND “english”[Language] AND “humans”[MeSH Terms]) NOT “Review”[Publication Type])

**Table 2:** Inclusion and exclusion criteria.

Inclusion Criteria (IC)	Exclusion Criteria (EC)
IC1: Articles that propose EML models applied to brain diseases. IC2: Articles that include the usage of ETs in ML models applied to brain diseases. IC3: Articles that include metrics to evaluate the ETs applied in brain diseases. IC4: Articles that respond to at least one of the previously formulated research sub-questions. IC5: Articles that show explicit numerical results. IC6: Articles that study a brain disease indirectly but are focused on its diagnosis, prognosis, risk, or treatment.	EC1: Articles that do not study human brain diseases, are oriented towards studying aspects other than the disease itself, or do not use actual data (use synthetic data). EC2: Articles that do not study, explore, explain, or integrate explainability as the central aspect of study or that do not use ETs. EC3: Articles that do not use classic ML models [54,55]. EC4: Articles outside of 2014–2023. EC5: Articles that are not in English.

**Table 3:** Types of ML models applied to brain diseases.

Id	Model	Count	Reference
ML01	Artificial Neural Network (ANN) ^1^	95	[58,59,60,61,62,63,64,65,66,67,68,69,70,71,72,73,74,75,76,77,78,79,80,81,82,83,84,85,86,87,88,89,90,91,92,93,94,95,96,97,98,99,100,101,102,103,104,105,106,107,108,109,110,111,112,113,114,115,116,117,118,119,120,121,122,123,124,125,126,127,128,129,130,131,132,133,134,135,136,137,138,139,140,141,142,143,144,145,146,147,148,149,150,151,152]
ML02	Support Vector Machine (SVM)	44	[58,62,63,64,68,69,74,76,77,80,82,83,87,88,90,91,92,96,99,100,111,113,121,122,123,130,132,133,139,144,149,150,153,154,155,156,157,158,159,160,161,162,163,164]
ML03	Random Forest (RF)	43	[68,69,74,77,80,82,87,91,92,96,99,100,102,113,122,123,142,143,144,149,150,154,155,157,158,160,162,163,164,165,166,167,168,169,170,171,172,173,174,175,176,177,178]
ML04	Gradient Boosting Machine (GBM) ^2^	41	[62,68,80,82,84,91,96,99,100,113,122,130,132,133,142,143,144,150,153,154,157,158,159,160,161,163,164,165,166,167,168,173,175,179,180,181,182,183,184,185,186]
ML05	Logistic Regression (LoR)	27	[63,68,87,92,96,99,121,122,123,129,130,131,132,133,143,144,150,153,154,155,156,157,158,159,160,167,187]
ML06	Decision Trees (DT)	18	[75,82,91,99,122,133,144,150,151,155,160,162,163,164,169,188,189,190]
ML07	K-Nearest Neighbors (K-NN)	12	[62,68,82,143,144,150,153,154,155,156,165,166]
ML08	Naive Bayes (NB)	11	[122,130,143,144,150,153,155,157,159,166,168]
ML09	Bootstrap Aggregating (BA)	2	[144,168]
ML10	Linear Discriminant Analysis (LDA)	2	[122,144]
ML11	Quadratic Discriminant Analysis (QDA)	1	[122]

^1^ ANN: this model includes Multi-layer Perceptron (MLP), Fully Connected Neural Networks (FCNN), Convolutional Neural Networks (CNN), Recurrent Neural Networks (RNN), Graph Neural Networks (GNN), and Hypergraph Neural Networks (HGNN). ^2^ GBM: this model includes eXtreme Gradient Boosting (XGBoost), Light Gradient Boosting Machine (LGBM), Categorical Boosting (CatBoost), Gradient Boosting Decision Tree (GBDT), and Adaptive Boosting (AdaBoost).

**Table 4:** ETs applied in the study of brain diseases.

Id	Explainability Technique	Count	Reference
ET01	Shapley Additive Explanations (SHAP)	43	[68,69,74,82,84,96,99,100,105,109,113,129,130,132,143,144,150,153,155,157,158,159,160,161,163,165,166,167,168,170,172,173,174,175,176,177,178,180,182,183,184,185,187]
ET02	Class Activation Map (CAM)	29	[58,59,64,75,76,79,81,90,93,94,95,98,103,104,106,109,110,115,116,117,119,122,126,127,134,137,139,140,148]
ET03	Local Interpretable Model-Agnostic Explanations (LIME)	15	[61,62,71,80,86,112,117,139,155,158,160,163,166,175,179]
ET04	Layer-Wise Relevance Propagation (LRP)	10	[73,76,107,118,125,135,141,142,145,146]
ET05	Saliency Maps (SM)	8	[66,70,73,76,92,108,135,138]
ET06	Attention Mechanism (AM)	5	[83,108,128,131,142]
ET07	Rules-based explanations (RBE)	4	[65,147,174,189]
ET08	t-distributed stochastic neighbor embedding (t-SNE)	3	[67,102,120]
ET09	Occlusion Sensitivity (OS)	3	[77,127,152]
ET10	Backpropagation (BP)	2	[72,106]
ET11	Partial Dependence Plots (PDP)	1	[164]
ET12	Permutation Feature Importance (PIMP)	1	[166]
ET13	Others	27	[60,63,72,78,85,88,89,91,97,101,111,114,117,121,123,124,133,136,139,149,151,154,156,162,171,181,186]

**Table 5:** Uses of EML models in brain diseases.

Disease	Study Type	Count	Reference
Alzheimer’s Disease (AD)	Diagnosis	43	[60,62,63,69,72,73,75,76,78,82,83,84,85,86,88,91,93,97,98,101,102,104,105,107,109,114,116,124,125,136,140,150,152,155,158,168,169,172,174,177,182,183,186]
	Prognosis	11	[60,87,110,111,117,123,157,158,171,172,174]
	Measurement	2	[149,151]
	Understanding	3	[61,89,127]
	Risk	2	[99,184]
	Conversion	4	[79,94,103,175]
Parkinson’s Disease (PD)	Diagnosis	13	[65,66,71,95,108,112,118,120,122,134,146,162,187]
	Prognosis	1	[163]
	Treatment	1	[156]
	Measurement	1	[176]
	Understanding	1	[141]
Brain Tumor (BT)	Diagnosis	5	[58,64,106,114,119]
	Prognosis	3	[113,121,164]
	Treatment	1	[154]
	Measurement	2	[80,128]
	Understanding	1	[170]
Acute Stroke (AS)	Diagnosis	1	[139]
	Prognosis	7	[100,130,132,144,159,165,189]
	Measurement	1	[126]
	Risk	2	[68,179]
Autism Spectrum Disorder (ASD)	Diagnosis	5	[90,92,115,133,161]
	Understanding	4	[89,135,138,188]
Multiple Sclerosis (MS)	Diagnosis	3	[77,81,160]
	Prognosis	2	[181,185]
	Understanding	1	[180]
Cerebral Hemorrhage (CH)	Diagnosis	2	[59,148]
	Prognosis	1	[143]
	Conversion	1	[153]
Epilepsy (EP)	Diagnosis	2	[67,147]
	Risk	1	[137]
Amyotrophic Lateral Sclerosis (ALS)	Diagnosis	1	[74]
	Understanding	2	[74,173]
Brain Inflammatory Diseases (BID)	Diagnosis	1	[119]
	Understanding	1	[166]
Acute Neurological Injury (ANI)	Diagnosis	1	[96]
	Prognosis	1	[96]
Brain Metastases (BM)	Conversion	2	[129,167]
Intracranial Pressure (ICP)	Risk	1	[70]
Neurodegenerative Diseases (NDD)	Diagnosis	1	[119]
Progressive Supranuclear Palsy (PSP)	Understanding	1	[127]
Cerebrovascular diseases (CVD)	Diagnosis	1	[119]
Cerebral Vasospasm (CVA)	Conversion	1	[178]
Schizophrenia (SZ)	Diagnosis	1	[142]
Hippocampal Sclerosis (HS)	Diagnosis	1	[145]
Dystonia (DY)	Understanding	1	[190]

## Supplementary Materials

The following supporting information can be downloaded at: https://www.mdpi.com/article/10.3390/neurolint16060098/s1, Table S1. PRISMA 2020 for Abstracts Checklist, Table S2. PRISMA 2020 Checklist.
